# Factors associated with stunting in healthy children aged 5 years and less living in Bangui (RCA)

**DOI:** 10.1371/journal.pone.0182363

**Published:** 2017-08-10

**Authors:** Pascale Vonaesch, Laura Tondeur, Sébastien Breurec, Petula Bata, Liem Binh Luong Nguyen, Thierry Frank, Alain Farra, Clotaire Rafaï, Tamara Giles-Vernick, Jean Chrysostome Gody, Ionela Gouandjika-Vasilache, Philippe Sansonetti, Muriel Vray

**Affiliations:** 1 Unité de Pathogénie Microbienne Moléculaire, Institut Pasteur, Paris, France; 2 Unité d'épidémiologie et d'expertise des maladies émergentes, Institut Pasteur, Paris, France; 3 Laboratoire de Microbiologie Clinique et Environnementale, Centre Hospitalo-Universitaire, Pointe-à-Pitre/Les Abymes, Guadeloupe, France; 4 Laboratoire de Bactériologie médicale, Institut Pasteur de Bangui, Bangui, République Centrafricaine; 5 Université des Antilles, Faculté de Médecine, Pointe-aà-Pitre, Guadeloupe, France; 6 Complexe Pédiatrique de Bangui, Bangui, République Centrafricaine; 7 Laboratoire des Virus entériques/Rougeole, Institut Pasteur de Bangui, Bangui, République Centrafricaine; 8 Unité d’épidémiologie des maladies infectieuses, Institut Pasteur de Dakar, Dakar, Sénégal; Institut de recherche pour le developpement, FRANCE

## Abstract

Stunting remains a major public health concern worldwide. Although its global prevalence is slowly decreasing, the actual number of affected children is still rising in Sub-Saharan Africa. In the Central African Republic (CAR), about one third of all children below the age of five are stunted. Stunting is correlated with many long-term consequences, including poor cognitive development and a higher rate of morbidity and mortality, making stunting a major contributor to poverty. In CAR, little is known about the factors that contribute to stunting. This study aimed at analysing, in a cross-sectional study, the main factors associated with stunting in a group of 414 children recruited between December 2011 and November 2013, aged five years or less and living in Bangui. For all children, demographic, socio-economic and anthropometric data were recorded and asymptomatic enteropathogen carriage was assessed in stool samples using classical microbiological assays. The study group had a mean age of 14.2±10 months. Fifty-eight percent (292/414) were boys, and 36 percent (148/414) exhibited stunted growth. Of the stunted children, 51% (75/148) showed a moderate delay in linear growth for their age group [height-for-age z-score (HAZ) between -2 and -3 SD] while 49% (73/148) presented a severe delay (HAZ < -3). Factors significantly associated with stunting included gender (aOR: 1.67; 95% CI: 1.07; 2.62 for boys compared to girls) and age (aOR of 3.98 (95% CI: 2.45; 6.46) for toddlers and aOR 4.42 (95% CI: 2.36; 8.28) for children compared to infants). Most importantly, we identified being overweight [weight-for-height z-score (WHZ) > 2 SD; aOR: 3.21; 95% CI: 1.50; 6.90 of overweight compared to normal weight] as also being significantly associated with stunting. This is the first study showing that even in the poorest countries of the world there is an association of stunting with being overweight.

## Introduction

Globally, one out of four children (25%) under five years of age experiences developmental and growth delays (stunting). Of these stunted children, 90% live in Sub-Saharan Africa and Asia (Levels and Trends in Child Malnutrition, WHO, UNICEF, World Bank, 2012). Stunting leads to deleterious effects on the child’s short-term and long-term health, including increased susceptibility to infection and impaired brain development [1–6]. A meta-analysis of 53 767 children living in Latin America, Asia and Africa showed more than a three-fold higher mortality in stunted compared to well-nourished children [7]. In Central African Republic (CAR), where this study was carried out, the percentage of stunted children under 5 years of age is alarmingly high at 41–43% (The World Bank and Global Nutrition report, data 2010). Stunting is a complex condition that may reflect several aetiologies, such as suboptimal breastfeeding in the first months of life, a poor and unbalanced diet and/or insufficient vitamin and/or micronutrient intake thereafter. Indirect factors that may also affect healthy growth include access to healthcare, education, wealth, political stability, social support networks, urbanisation and living conditions. The influence of both direct and indirect factors was reviewed recently in the WHO Conceptual Framework on Childhood stunting and is summarized in Dewey *et al*, 2011 [5].

Stunting in developing countries often starts *in utero* and its severity increases until it reaches a plateau at about two years of age, a time period called the “1000 days” [8,9]. Notably, a recent meta-analysis of 42 studies showed that complementary feeding practices to overcome chronic undernutrition were at best able to correct for roughly a third of the encountered growth deficits [10]. This finding suggests that other factors may be implicated in stunting. Factors that have been linked to stunting include poor hygiene and sanitation and recurrent gastrointestinal infections [4,11–17].

However, little data exists on the factors associated with stunting in central Africa, mostly due to recurrent political instabilities that make data collection challenging. For the CAR, we could find only a single study on risk factors associated with stunting. This study used data collected in a demographic health survey in the year 2000 and included 12 949 children aged 0–59 months. Poor socio-economic status of the family was found as the main risk factor for stunting [18]. Given the little information we have on risk factors of stunting in the CAR and its high prevalence of stunting, there is an urgent need to further investigate the factors underlying linear growth failure in this part of the world in greater detail in order to design better prevention and intervention strategies.

Stunted children are more susceptible to infections, particularly diarrhoeal and respiratory diseases [19] as well as malaria [10,20]. Infections enhance undernutrition, thus creating a vicious cycle leading to growth defects. In different animal models, it was shown that malnourished mice experimentally infected with *Cryptosporidium* spp. [21], entero-aggregative *Escherichia coli* [22,23], *Giardia lamblia* [24], or a cocktail of different non-pathogenic faecal bacteria [25] showed signs of enteropathy and displayed delayed growth.

Similarly, a longitudinal study performed on 197 children aged 2 to 48 months in rural Bangladesh in 1978 and 1979 revealed an association of *Shigella*-mediated diarrhoea and subsequent delays in linear growth (0.055 cm less growth/percent days, P = 0.008) while no association was found with enterotoxic *E*. *coli*-mediated diarrhoea and subsequent linear growth of infected children [26]. Another longitudinal study conducted between 1989–1991 on *Cryptosporidium parvum* in a cohort of 185 children aged 0–3 months living in Lima (Peru) followed for two years revealed a significant negative association between linear growth and *Cryptosporidum parvum* infection as well as the efficiency of catch-up growth [27], while another longitudinal study conducted on 545 children followed from birth to three years of age in a rural coastal region of Kenya in 2007–2010 showed a positive association between infection with *Ascaris sp*., soil-transmitted helminths, *Giardia and* malaria and linear growth failure (decreased height when infected with *Ascaris* at month 24: -0.93 cm, p<0.001, with soil transmitted helminths at month 24–0.35cm, p = 0.01, with *Giardia spp*. at month 12–0.5 cm, p = 0.003 and with malaria at month 18: -0.63 cm, p = 0.003). However, these associations were not found in all age groups tested, indicating a complex interplay between infection and subsequent stunting [28]. Notably, because of reciprocal exacerbation of undernutrition and infection, it has been difficult to distinguish between cause and effect in these epidemiological studies. In addition, many of these epidemiological studies suffer from a lack of follow up studies to confirm and consolidate the results. The direct effect of pathogens on child stunting therefore still remains poorly understood.

Interestingly, there is growing evidence that children not suffering from diarrhoea asymptomatically carry diarrhoeal pathogens [29,30,31]. The Torcadia study, a matched case-control study performed between November 2011 and December 2013 in Bangui, the capital of Central African Republic [30], sought to determine the most important pathogens in severe childhood diarrhoea leading to hospitalization among children under five years old. A considerable number of asymptomatic pathogen carriage was observed among the group of non-diarrheal children [30]. However, this data set was not analysed for risk factors associated with stunting and whether there is a relationship between this asymptomatic enteropathogen carriage and stunting. Indeed, a recent study suggests that asymptomatic pathogen carriage may be associated with stunting [32].

In this study, we performed a secondary data analysis on all healthy children included in the Torcadia study. This cross-sectional study aimed to determine the risk factors associated with stunting in children under 5 years of age living in Bangui, the capital of CAR. The secondary objective was to assess if asymptomatic pathogen carriage is associated with stunting in seemingly healthy children.

## Material and methods

### Data source

**Fig 1:**
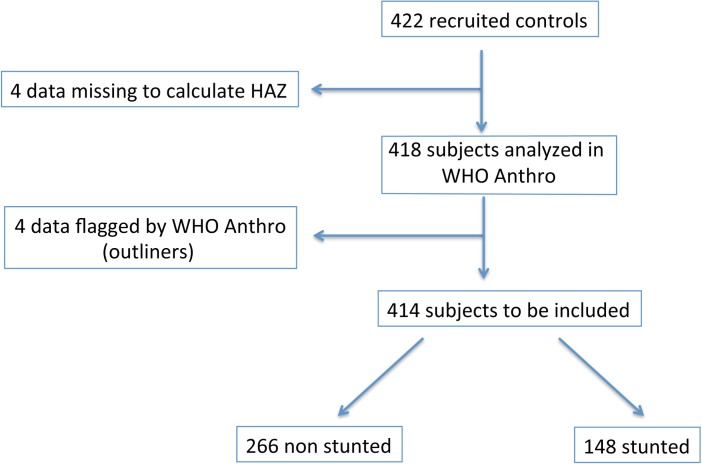
Flow-chart of the subjects included in the study.

This was a cross-sectional study on all children not suffering of diarrhoea (n = 422, non hospitalized, healthy children) included in the Torcadia study, a study conducted in Bangui between November 2011 and December 2013 [30]. The inclusion criteria of these children were the following: (1) aged between 0–59 months; (2) no history of diarrhoea or antibiotics in the 7 days prior to inclusion; (3) in good general health; (4) recruited in the community; and (5) written consent by the legal representative to participate in the study. Exclusion criterion was a caregiver’s spontaneous declaration of HIV-positive status of their children. The children were studied at their homes and samples were collected and questionnaires administered by a dedicated study nurse. All healthy children with a valid height-for-age z-score (n = 414) were included in the final analysis (see flow-chart in Fig 1).

### Ethics statement

The initial study protocol was approved by the Comité de Recherche Clinique of the Institut Pasteur and the National Committee of the Central African Republic. The protocol included written consent for participation in the study and subsequent use of the samples and data was requested from all parents or guardians of any child participant on their behalf.

### Collected variables

The collected variables include anthropometric measurements (weight, height/length, mid-upper arm circumference (MUAC), and head circumference), age, gender, family structure, different indicators of socioeconomic status (profession of parents, working situation, household size, description of living conditions, general luxury goods, mobility…), different indicators of sanitary status (drinking water, eating with fingers, access to sanitary facilities, …), and S2 Table for pathogen list).

Anthropometric variables were defined according to the WHO Growth Child standards 2006 [33,34] and calculated using the World Health Organization WHO Anthro software (version 3.2.2, January 2011). The following variables were calculated: height-for-age z-score (HAZ), Weight-for-age z-score (WAZ), BMI-for-age z-score (zBMI), Weight-for-height z-score. The following cut-offs as defined by the WHO were used: stunted: < -2 HAZ (moderately stunted: -3 ≤ HAZ < -2; severely stunted: HAZ < -3); acute malnutrition based on WHZ score: < -2 WHZ (acute malnutrition: -3 ≤ WHZ < -2; severe acute malnutrition: WHZ < -3). The definition of being overweight was based on zBMI (Normal weight: -2 SD≤ zBMI≤ 2SD, Overweight: 3≤ zBMI > 2 SD and obese zBMI > 3 SD), as this measure was shown to be a good predictor of actual body fat [35]. As to facilitate the multivariate analysis WHZ was used to indicate both, under- and overnutrition, in the logistic regression.

The microbiological analyses used included the following detection methods of pathogen detection of stool samples: *Shigella* spp. and *Salmonella enterica*, on Hektoen Enteric agar (Bio-Rad, Marnes-la-Coquette, France), *Escherichia coli* and other Gram-negative bacteria on bromocresol purple lactose agar and Levine's eosin-methylene blue agar (Bio-Rad, Marnes-la-Coquette, France), *Yersinia enterocolitica* on Cefsulodin-irgasan-novobiocin agar (Bio-Rad, Marnes-la-Coquette, France), and *Vibrio cholerae* on thiosulfate—citrate—bile salts—sucrose agar (Bio-Rad, Marnes-la-Coquette, France) after an initial selective enrichment with alkaline peptone water.

*E*. *coli* pathotypes were determined on a pooled sample from five putative *E*. *coli* colonies from every stool sample using a single-test multiplex PCR as previously described [36]: typical EPEC (*bfpB* positive), atypical EPEC (*escV* positive, *bfp* negative, *stx* negative), STEC *(escV* positive/negative, *bfp* negative, *stx1* positive, *stx2* positive, or both), ETEC (*elt* positive, *estIa* positive, *estIb* positive), EIEC (*invE* positive) and EAEC (*aggR* positive or *astA* positive with pic positive). The *E*. *coli*-specific *uidA* gene was used to confirm that the collected colonies were indeed *E*. *coli*. DNA from faecal samples was extracted using the QIAamp DNA Stool Mini Kit (Qiagen, Courtaboeuf, France). To improve assessment of the involvement of *Shigella*, a PCR assay based on amplifying the invasion plasmid antigen H (*ipaH*) gene contained in EIEC and *Shigella* spp was performed.

For the detection of group A rotaviruses, astroviruses and adenoviruses, the ProSpecT kits (Oxoid, Thermo Fisher Scientific, Basingstoke, UK) were used. To detect noroviruses of the genogroups (GG) I and II, the IDEIA Norovirus kit (Oxoid, Thermo Fisher Scientific, Basingstoke, UK). All kits were used according to the manufacturer’s instructions.

For the parasitic analyses, a subsample of the stools was concentrated by the merthiolate iodine formaldehyde concentration technique and examined for helminth eggs and protozoa cysts. Differentiation of pathogenic *Entamoeba histolytica* from non-pathogenic *Entamoeba dispar* was performed by enzyme-linked immunosorbent assay (ELISA) (Fumouze Diagnostics, France). Multiplex PCR was used for the detection of *Cryptosporidium hominis* and *Cryptosporidium parvum* on extracted DNA from feces. For a detailed description of the microbiological detection methods used, see reference [30] and [36].

### Statistical analyses

The statistical analysis was performed with Stata 13. Significance level was fixed for all analyses at 0.05 and all tests performed were bilateral. Quantitative variables were expressed as mean (± Standard Deviation), or median (interquartile range); qualitative variables were expressed as percentage. The stunted vs. non-stunted groups were compared using Chi2 or Fisher Exact test for qualitative variables and the Student t Test or the Mann-Whitney U test for quantitative variables. Factors potentially associated with stunting in univariate analysis with a p-value of <0.25 were included in a backward logistic regression. Results are reported as adjusted OR with 95% CI, corrected for age, gender, weight-for-height z-score and culture-based bacterial carriage.

## Results

### General characteristics of participants

The majority of the population studied lived in an urban setting (392/414; 95%). The general characteristics of the study population is given in Table 1, anthropometric characteristics are given in Table 2. The study group had a mean age of 14.2±10 months, the youngest child being one month old, and the oldest 58 months. Fifty-eight percent (240/414) of the study population were boys.

**Table 1:** Description of socioeconomic characteristics (n = 414).

Description of study population: socioeconomic status, sanitation	
Socio-economic status of family*	
Lowest income	34/414 (8%)
Middle income	331/414 (80%)
Highest income	49/414 (12%)
**Mother completed at least primary school**	224/414 (54%)
**Father working**	318/414 (77%)
**Drinking water source of children**	
At least sometimes water from well	81/414 (19%)
Running water or from fountain only	222/414 (54%)
Only mineral water	91/414 (22%)
Other (breastfeeding only etc.)	20/414 (5%)
**Drinking water source of family**	
At least sometimes water from well	239/414 (58%)
Running water or from fountain only	174/414 (42%)
Only mineral water	0/414 (0%)
**Family treats water by chlorination**	128/414 (31%)
**Eating with**	
Fingers only	113/414 (27%)
Cutlery only	195/414 (47%)
Both	97/414 (24%)
No data	9/414 (2%)

*defined as in Breurec et al., Plos Neglected Tropical Diseases 2015

**Table 2:** Description of general study population: Anthropometric measurements (n = 414).

Description of study population: age, gender, anthropometric measurements	
**Females**	122 /414 (46%)
**Age of study population (months)** ^**1**^	14.2 ±10.0
**HAZ of study population**^**1**^^,^ ^**2**^	-1.45 ±1.7
**Stunted (based on HAZ)**	
Normal growth (HAZ ≥ -2 SD)	266/414 (64%)
Stunted (HAZ < -2 SD)	148/414 (36%)
**WHZ of study population**^**1**^^,^ ^**3**^	0.25 ± 1.31
**Acute malnutrition (based on WHZ)**	
Normal weight (-2 SD≤ WHZ≤ 2SD)	363/414 (88%)
Acute malnutrition (WHZ < -2 SD)	16/414 (4%)
**BMI z-score of study population**^**1**^^,^ ^**4**^	0.38 ±1.37
**Overweight (based on zBMI)**	
Normal weight (-2 SD≤ zBMI≤ 2SD)	355/414 (86%)
Overweight and obese (zBMI >2 SD)	42/414 (10%)

^1^ Mean ± Standard Deviation

^2^ stunted: < -2 height-for-age z-score (HAZ) (moderately stunted: -3 ≤ HAZ < -2; severely stunted: HAZ < -3)

^3^ acute malnutrition based on weight-for-height z-score (WHZ) score: < -2 WHZ (moderately acutely malnourished (MAM): -3 ≤ WHZ < -2; severely acutely malnourished (SAM): WHZ < -3).

^4^ Overweight based on Body-Mass Index z-score (zBMI): overweight: zBMI> 2SD (Overweight: 3≤ zBMI > 2 SD and obese zBMI > 3 SD)

Surprisingly, only 3% (14/414) of the parents co-habited; instead, most mothers were living with their children together with their extended family (397/414). Less than a third of the mothers declared themselves to be working (125/414). The median number of family members was 7 (interquartile range 5–10) and the median number of children below the age of 5 in each household was 2 (interquartile range 1–3). Eighty-eight percent (365/414) of all families had access to improved water, as defined by either running water or water from a public fountain. Seventy-six percent (313/414) of the children were given exclusively improved water for drinking. For one-fifth of the children (91/414), bottled mineral water was reported by the caregiver as the sole drinking water. It is noteworthy that the children received different water for drinking from that used by the family. Roughly a third of all families treated drinking water by chlorination (128/414).

### Anthropometric characteristics of the participants

Thirty-six percent (148/414) of the study population showed stunted growth, of which roughly half showed a moderate delay (height-for-age z-score (HAZ) between -2 and -3 SD) and half a severe delay (HAZ < -3) in linear growth for their age group. Stunting was higher in boys, with 97/241 (40%) of all boys being stunted compared to 55/177 (31%) of girls. The stunted group had a higher median age (median 15.5 months, interquartile range: 11–22 months) than that observed for the non-stunted group (median 9 months, interquartile range: 7–13 months). The mean HAZ drastically decreases with increased age, averaging -0.89 HAZ (SD 1.44) for infants (0–11 months), -1.96 HAZ (SD 1.77) for toddlers (12–23 months) and -2.33 HAZ (SD 1.73) for children (≥ 24 months), while the mean weight-for-height z-score (WHZ) remained relatively stable (-0.46 (1.11 SD); -0.63 (1.17 SD); -1.2 (1.21 SD)) in infants, toddlers and children, respectively. The percentage of stunted children hence increased with age, progressing from 21% (45/218) in infants, 52% in toddlers (72/138) to 53% in children (31/58).

An analysis of the weight category, based the body weight index (zBMI), in relation to the stunting phenotype yielded the following: 356 of 414 children (86%) were of normal weight. Of these, 32% (120/372) were concurrently stunted. 16 of 414 children (4%) were underweight. Of these, 19% (3/16) were also concurrently stunted. In contrast, 42 of 414 (10%) were overweight, and 67% (28/42) of these were also concurrently stunted.

Thus, a striking percentage of overweight children were concurrently stunted. These overweight and concurrently stunted children accounted for 7% (28/414) of the entire study population,

### Asymptomatic pathogen carriage as a risk factor for stunting

**Fig 2:**
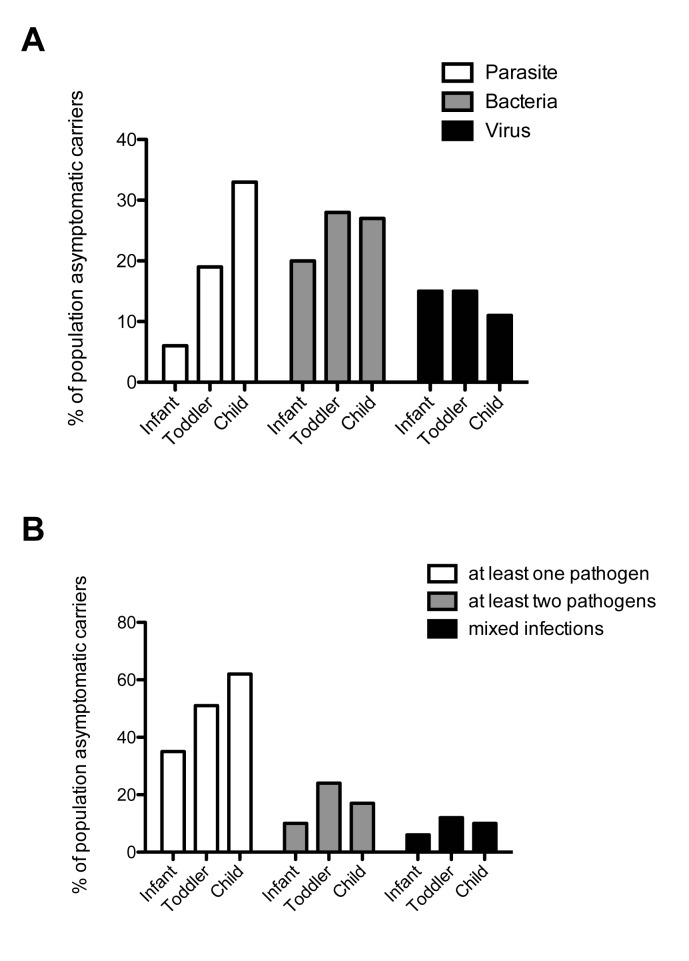
Pathogen load in different age categories. **A:** Pathogen load by age category for the three main groups of pathogens, parasites (white bars), bacteria (grey bars) and viruses (black bars). **B**: Infection with multiple pathogens by age category. White bars indicate the presence of at least one pathogen of any group (parasite, bacteria or virus), grey bars the presence of at least two pathogens of any group (parasite, bacteria or virus) and black bars indicate mixed infections with at least one representative of two different groups (virus, parasite or bacteria) in the same child. Infant: 0–11 months, Toddler: 12–23 months; Child: ≥ 24 months).

Overall asymptomatic pathogen carriage, as measured by conventional microbiological techniques, was generally low (see Table 3 and S2 Table for a full list). Only 14% (57/414) of the children tested positive for one or more parasites, 16% (61/414) of the children tested positive for one or more viruses, and only 23% (97/414) of children tested positive for a pathogenic bacterium. Of these bacterial pathogens, 9% (33/414) were culture positive. Cumulative, 44% (177/414) of children tested positive for at least one pathogen. Co-infection of at least two pathogens occurred in 16% (66/414) of children, while mixed infections of at least two groups of pathogens (bacteria, virus and/or parasite) occurred in 9% (37/414) of children. Asymptomatic pathogen carriage increased with age (see Fig 2). The most prevalent parasites, with about 1/10 children infected, were *Cryptosporidium* and *Giardia*, while only 1/50 children showed amoeba in their stools. Bacteria were more readily detected by PCR, as illustrated through the increased detection of *Shigella* in PCR compared to culture techniques. The most prevalent bacteria detected were *Shigella* and pathogenic *E*. *coli* (around 1/10 children), the most prevalent single *E*. *coli* strain, with around 1/20 carrier children, was enterotoxic *E*. *coli* (ETEC). About 1/20 children carried either rotavirus, norovirus, adenovirus or astrovirus (see S2 Table).

**Table 3:** Description of general study population for asymptomatic pathogen carriage (n = 414).

**Combined scores**	
**At least one pathogen detected**	177/414 (44%)
**At least two pathogens detected**	66/414 (16%)
**Mixed infection with at least two groups of pathogens (virus, parasites, bacterium)**	37/414 (9%)
**Parasites**	
**At least one parasite detected by PCR or microscopy**	57/414 (14%)
***Cryptosporidium parvum/hominis* (PCR)**	39/414 (9%)
***Giardia intestinalis* (microscopy)**	32/414 (8%)
**Amiba (microscopy)**	7/414 (2%)
**Bacteria**	
**At least one bacterium detected by culture or PCR**	97/414 (23%)
**At least one bacterium detected by culture**	33/414 (6%)
***Shigella spp*. *(PCR)***	35/414 (8%)
***Shigella spp*. *(culture)***	4/414 (1%)
***Salmonella spp*. *(culture)***	13/414 (3%)
**At least 1 pathogenic *E*. *coli* by PCR**	49/414 (12%)
**ETEC**	19/414 (5%)
**Virus**	
**At least one virus detected**	61/414 (16%)

There was a significant association between carriage of a parasite or a bacterium detected in culture and stunting in the univariate analysis (p = 0.022) (see Table 4). After adjustment for either age alone (data not shown) or age and gender (data not shown), no statistically significant association between parasite carriages and stunting was observed. A trend towards association between bacterial carriage and stunting was observed for the 9% of bacterial pathogens identified by culture methods, although the association remained non-significant (p = 0.069) (see Table 4). Notably, this trend is independent of age group as all age categories displayed an unadjusted OR of ~2, suggesting that significance might not have been reached due to a limited sample size.

**Table 4:** Risk factors associated with stunting (n = 414).

	Non stunted	Stunted	Unadjusted OR	p-value unadjusted	Adjusted OR	p-value adjusted OR*
	N = 266	N = 148	(95%CI)	OR	(95%CI)*	
**General factors**						
**Females**	122 (46%)	52 (35%)	0.64 (0.42; 0.97)	0.034	0.61 (0.38; 0.94)	0.027
**Age (months)**^**1**^	12.2 ± 9.5	17.6 ±10.1		p< 0.0001		p< 0.0001
Infant	173 (65%)	45 (30%)	1		1	
Toddler	66 (25%)	72 (49%)	4.19 (2.63; 6.70)		3.98 (2.45; 6.46)	
Child	27 (10%)	31 (21%)	4.41 (2.40; 8.13)		4.42 (2.36; 8.28)	
**Weight-for-height z-score (WHZ)**^**1**^	0.08 ±1.26	0.55 ±1.34		0.003		0.009
Normal	239 (90%)	124 (84%)	1		1	
MAM/SAM	13 (5%)	3 (2%)	0.44 (0.12; 1.59)		0.67 (0.18; 2.54)	
Overweight	14 (5%)	21 (14%)	2.89 (1.42; 5.88)		3.21 (1.50; 6.90)	
**Sanitation and socio-economic factors**						
**Water source of child**				0.010		0.498
At least sometimes water from well	46 (17%)	35 (24%)	1			
Running water or from fountain only	136 (51%)	86 (58%)	0.35 (0.18; 0.68)			
Only pure water	72 (27%)	19 (13%)	0.83 (0.50; 1.39)			
Other (breastfeeding etc.)	12 (5%)	8 (5%)	0.88 (0.32; 2.37)			
**Eating with**				0.0005		0.741
Fingers only	60 (23%)	53 (37%)	1			
Cutlery only	144 (55%)	51 (35%)	2.07 (1.24; 3.46)			
Both	56 (22%)	41 (28%)	2.49 (1.53; 4.06)			
**Mother lives with family**	258 (97%)	139 (94%)		0.139		0.175
**Mother completed at least primary school**	141 (53%)	83 (56%)		0.508		0.526
**Socio-economic score**^**2**^				0.346		0.085
Lowest income	19 (7%)	15 (10%)	1		1	
Middle income	212 (80%)	119 (80%)	0.71 (0.35; 1.45)		0.55 (0.25; 1.23)	
Highest income	35 (13%)	14 (10%)	0.51 (0.20; 1.27)		0.31 (0.11; 0.88)	
**Asymptomatic pathogen carriage**						
**Pathogen found**	106 (40%)	71 (49%)		0.119		0.805
**(Parasite, Bacterium or Virus)**						
**Parasite found**	29 (11%)	28 (19%)	1.91 (1.09; 3.35)	0.025		0.370
***Giardia lamblia***	17 (6%)	15 (10%)		0.175		0.801
**Virus found (PCR)**	41 (15%)	20 (14%)		0.384		0.390
**Rotavirus (PCR)**	13 (5%)	3 (2%)		0.151		0.148
**Noroviurs (PCR)**	11 (4%)	3 (2%)		0.253		0.253
**Bacterium in culture found**	17 (6%)	18 (12%)	2.32 (1.13; 4.75)	0.022	2.04 (0.95; 4.41)	0.069
***Salmonella* spp.**	6 (2%)	7 (5%)		0.176		0.586
**ETEC**	8 (3%)	11 (7%)	2.59 (1.02; 6.59)	0.046		0.299

* OR adjusted for age category, gender, weight-for-height z-score, positive for bacterial culture

^1^ Mean ± standard deviation

^2^ as described in Breurec et al., PNTD 2016

### Other risk factors for stunting

Table 4 shows the prevalence of stunting and its associated risk factors. In a univariate analysis, several factors indicating hygiene status. For example, providing bottled mineral water as the child’s sole water source or using cutlery for eating were associated with less stunting. However, the association disappeared when adjusted for age. Education of mother and size of the household were not significantly associated with stunting. Socio-economic status was not significantly associated (p = 0.085), showed however a trend to protection as the socio-economic status rose with an aOR of 0.55 (95% CI: 0.25; 1.23) from lowest income to middle income and an aOR of 0.31 (95% CI: 0.11; 0.88) from lowest to highest income (see Table 4). Using logistic regression, the only three variables independently associated with stunting were gender, age and being overweigh. Boys showed an approximately 1.7x higher risk than girls of being stunted (aOR: 1.67; 95% CI: 1.07; 2.62). Toddlers (12–23 months) were about 4-fold (aOR 3.98; 95% CI: 2.45; 6.46) more likely to be stunted compared to infants (0–11 months), while children (24–59 months) were about 4.5-fold (aOR 4.42; 95% CI: 2.36; 8.28) more likely to be stunted than infants. In addition, overweight children were roughly three times more likely to be stunted (aOR: 3.21; 95% CI: 1.50; 6.90) compared to normal weight children.

### Description of the stunted-overweight population

Stunted children showed a more than 4-fold increase (aOR: 4.2; 95% CI: 2.13; 8.27) in the risk of being overweight. Around 7% (28/414) of the total study population was concurrently stunted and overweight (stunted-overweight). The characteristics of this population, as well as the stunted-only and overweight-only populations, is given in S3 Table. Similar to the entire stunted population, the risk of the stunted-overweight phenotype increased significantly with age (OR 3.48, 95% CI: 1.44, 8.36 in toddlers compared to infants; OR 3.5, 95% CI: 1.21, 10.08 in children compared to infants). No other significant associations with potential risk factors were identified.

## Discussion

This cross-sectional study analysed 414 children aged five years and under living in Bangui for risk factors associated with stunting. The children were recruited from December 2011 until November 2013, and thus before the outbreak of the 2013–16 civil war. After correction for age, we found no significant statistical association between asymptomatic pathogen carriage and stunting. However, an increased risk of stunted children concurrently being overweight was observed. This is one of the first studies in the CAR looking at risk factors associated with stunting and the first study to link stunting with a much higher increased risk of being overweight in one of the least developed countries of the world. Indeed, stunted children were much more likely to be overweight than underweight. Given this finding, nutritional supplementation to ameliorate the stunting phenotype, which is believed to be, at least in part, a manifestation of chronic undernutrition, need to be carefully designed and monitored in order to prevent exposing the stunted children to the additional health risks that are associated with being overweight.

Our study has however several weaknesses: first and foremost, stunting is a gradual process, emerging from a long-term chronic undernutrition and/or repeated infections (reviewed in [6]). It would therefore be better to analyse risk factors in a longitudinal study, in order to be able to draw causal conclusions. The study also did not include a large number of children, which made it impossible to further stratify the children based on age groups. As infants and toddlers experience a different environment than older children (e.g. eating with hands, receiving special drinking water and other foods), it would be interesting to be able to analyse the associated risk factors of stunting in the three age groups independently in future studies.

Molecular techniques were more sensitive to detect low levels of pathogens, as illustrated through the carriage of *Shigella*, which was assessed through both culture and PCR. It would therefore be interesting to use more sensitive techniques in future studies to look at asymptomatic pathogen carriage. However, given the long list of pathogens tested, our study contributes valuable insights into the possible associations between asymptomatic pathogen carriage and stunting. It also highlights the need to adjust for age when analysing the association in cross-sectional studies as pathogen carriage was associated with stunting in a univariate analysis but disappeared once adjusted for age.

Our analysis revealed age, gender and being overweight to be the most significant risk factors associated with stunting in young children living in the CAR. In a meta-study that consolidated 18 separate studies from Sub-Saharan Africa, the main factors associated with stunting were gender (boys being more stunted than girls), socio-economic factors and maternal education [37]. Another meta-analysis of 16 demographic and health surveys from Sub-Saharan Africa showed that boys of socio-economically weaker families were at greater risk of experiencing stunted growth than girls [38]. In accordance with these findings, our data identified boys to be at a higher risk than girls of being stunted. To date, it is unclear what social or biological reason might account for this difference. Our study did not find any association of stunted growth with socio-economic status or maternal education. This could be for several reasons: most of the families were living in similar conditions, which did not permit detection of a possible effect of socio-economic status on growth. Furthermore, because the study was conducted in the capital, formal education might have less influence on health, nutrition and sanitation choices than it would have in a rural setting, where access to information is more difficult.

Children in low-income countries are prone to helminth infections (see as an example [28,39–41], a phenomenon suspected to be potentially associated with chronic undernutrition [11,42]. It is surprising that no helminths were found in the stool specimens given the high burden of chronic malnutrition, the still wide-spread use of non-improved well water by 239 of the 414 families (58%) and the socio-economic challenges facing the people of the CAR,. This observation could be due to the frequent use of mebendazole, a drug that is acting mainly against helminthic infections and is widely available and used by mothers in Bangui to treat their children. In addition, mebendazole is given to children through mass deworming campaigns by non-governmental organizations. For example, in 2013, 417,898 children aged one to five years received mebendazole in CAR through a UNICEF program (https://www.unicef.org/appeals/files/HAC_2014_CAR_-_Revised_06-05-2014.pdf). More *in depth* data on this question would be needed in order to conclusively explain this phenomenon and to investigate the impact this will have on the health of these children.

Our analysis revealed that 7% of all children in the study group were concurrently stunted and overweight. This phenomenon was described as early as 1996 by Popkin and collaborators [43] among children aged 3–6 and 7–9 years in national surveys in Russia, Brazil, the Republic of South Africa and China, four countries entering a nutritional transition at this time. The risk ratios of being overweight for stunted children ranged from 1.7 to 7.8 in these studies. This observation gained momentum again in the last years due to the alarming global increase in childhood obesity and the particularly dramatic upsurge of obesity in developing countries [44]. Stunted preschool children were shown to have an increased risk to be overweight in South Africa (the Limpopo province)[45], Cameroon [46], Brazil [47], rural Mexico [48], Guatemala [49], Uruguay, Ecuador [50,51], China [52] and Indonesia [53]. Prevalence of concurrent stunting and being overweight ranged in these studies between 2.5% in China [52] to over 19% in the study conducted in the Limpopo region of South Africa [45]. There are several hypotheses that could explain the seemingly paradox nature of the co-existence of these two phenomenon. The first hypothesis, proposed by Mamabolo and colleagues [45], contends that poor food quality is to blame. Such food is typically low in animal proteins, micronutrients and fat and rich in carbohydrates. The authors hypothesize that the low protein and fat content leads to linear growth deficits while the high carbohydrate content leads to increased fat mass. Another hypothesis is the so-called Barker hypothesis, which emerged from observations during the Dutch Famine of the Second World War. These observations showed that men suffering from food deprivation during the first half of gestation had a higher risk of obesity at age 19 [54]. Further work suggested that food deprivation early on in life can lead to metabolic changes that pre-dispose for obesity and metabolic disease later in life [55–58]. A proposed explanation for the Barker hypothesis is that in contrast to normal weight gain, malnourished children have a disequilibrium in growth hormone and other growth factors, leading them to develop a higher proportion of fat and lower proportion of lean tissue. This explanation was supported in a study assessing the body composition in 20 stunted versus 30 healthy children aged 11 to 15 years and living in the slums of Sao Paulo, Brazil [59]. Furthermore, in a longitudinal study among school girls followed for two years, stunted girls gained more weight when exposed to a high fat diet compared to their non-stunted colleagues and also had higher central fat accumulation as reflected through a lower waist-to-hip ratio [60]. The same observation was made in another study where children were followed for over four years [61]. A study of 58 pre-pubertal adolescents also showed that fasting beta oxidation of fatty acids was decreased in stunted compared to healthy children living in a shantytown of Sao Paulo [62], while their resting energy expenditure remained normal [63]. Hence, the decreased ability to break down fatty acids to Acetyl-CoA while not changing the amount of energy used in the body in general could lead to enhanced presence of fatty acids and hence possibly leading to deposition of the fatty acids as stored fat tissue. The same investigators also showed that in a group of 56 children aged 8–11 years living in the shantytown of Sao Paulo, the regulation of energy intake was impaired in stunted children compared to the normally nourished control group, suggesting that part of the phenomenon could also be due to “opportunistic overeating” by the stunted children leading to excessive food intake and hence overweight [64].

More data is needed in order to really understand the metabolic mechanisms leading to the phenomenon of the concurrent occurrence of stunting and being overweight (termed stunting-overweight).

Our study is clearly underpowered to look at risk factors associated with this phenomenon. More studies in the CAR are needed to shed light on the precise risk factors and mechanisms underlying stunting-overweight.

As the CAR is one of the poorest countries in the world, our study reveals for the first time that stunting-overweight is no longer a problem of richer affluent communities such as Brazil, China or Indonesia undergoing large-scale nutritional transition, but represents a major public health risk for the entire developing world in the coming decades.

More data is needed in order to consolidate the findings described in this paper. However, the co-occurrence of stunting and overweight in one of the poorest countries of the world is alarming and, if the results are confirmed in further studies, the increased risk of stunted children for obesity should be considered when designing treatment schemes for chronically malnourished children.

## Supporting information

S1 Table Description of general study population: Additional anthropometric indicators: MUAC and zBMI as indicators of acute malnutrition and underweight, WHZ as indicator for obesity, (n = 414). (DOCX)

S2 Table Description of general study population: Asymptomatic pathogen carriage (n = 414). (DOCX)

S3 Table Description of the stunted-overweighted, stunted non-overweight and non-stunted overweight populations. (DOCX)

S4 Table Variables used in this study. (XLS)
