# Supplementary material for: Factors associated with stunting in healthy children aged 5 years and less living in Bangui (RCA)
Source: PLoS One. 2017 Aug 10;12(8):e0182363. doi: 10.1371/journal.pone.0182363 (PMC5552116; doi:10.1371/journal.pone.0182363)
Supplement: S1 Table — (DOCX) [file pone.0182363.s001.docx]

**S1 Table: Description of general study population: additional anthropometric indicators: MUAC and zBMI as indicators of acute malnutrition and underweight, WHZ as indicator for obesity, (n=414)**

| **MUAC of study population^1,^ ^2^  (cm)** | 138 ±13 cm |
| --- | --- |
| **Acute malnutrition based on MUAC^2^**  Normally nourished  Acute malnutrition | 355/414 (86%)  59/414 (14%) |
| **Underweight (based on zBMI)**  Underweight (zBMI < -2 SD) | 16/414 (4%) |
| **Overweight (based on WHZ)**  Overweight and obese (WHZ > 2 SD) | 35/414 (8%) |

^1^ Mean ± standard deviation

^2^ normally nourished: MUAC > 125 cm; MAM: 115 cm≥ MUAC ≥ 125 cm; SAM: MUAC< 115 cm
