# Supplementary material for: Factors associated with stunting in healthy children aged 5 years and less living in Bangui (RCA)
Source: PLoS One. 2017 Aug 10;12(8):e0182363. doi: 10.1371/journal.pone.0182363 (PMC5552116; doi:10.1371/journal.pone.0182363)
Supplement: S2 Table — (DOCX) [file pone.0182363.s002.docx]

**S2 Table: Description of general study population: asymptomatic pathogen carriage (n=414)**

| **Parasites and yeasts** | |
| --- | --- |
| **Microsporidia (microscopy)** | 0 (0%) |
| ***Cyclospora cayetanensis***  **(microscopy)** | 0 (0%) |
| ***Isospora belli* (microscopy)** | 1 (0.2%) |
| ***Cryptosporidium parvum/hominis* (PCR)** | 39 (9%) |
| ***Giardia intestinalis* (microscopy)** | 32 (8%) |
| **Amibes (microscopy)** | 7 (2%) |
| ***Candida* spp.** | 2 (0.5%) |
|  |  |
| **Bacteria** | |
| ***Vibrio cholera (culture)*** | 0 (0%) |
| ***Staphylococcus aureus (culture)*** | 0 (0%) |
| ***Plesiomonas shigelloides (culture)*** | 0 (0%) |
| ***Aeromonas hydrophila (culture)*** | 0 (0%) |
| ***Yersinia enterocolitica (culture)*** | 0 (0%) |
| ***Shigella spp. (PCR)*** | 35 (8%) |
| ***Shigella spp. (culture)*** | 4 (1%) |
| ***Salmonella spp. (culture)*** | 13 (3%) |
| **EIEC** | 6 (1%) |
| **STEC** | 1 (0.2%) |
| **ATEC** | 11 (3%) |
| **EPEC** | 0 (0%) |
| **ETEC** | 19 (5%) |

| **Virus (ELISA)** | |
| --- | --- |
| **Norovirus** | 14 (4%) |
| **Astrovirus** | 18 (5%) |
| **Adenovirus** | 20 (5%) |
| **Rotavirus** | 16 (4%) |
