# Supplementary material for: Factors associated with stunting in healthy children aged 5 years and less living in Bangui (RCA)
Source: PLoS One. 2017 Aug 10;12(8):e0182363. doi: 10.1371/journal.pone.0182363 (PMC5552116; doi:10.1371/journal.pone.0182363)
Supplement: S3 Table — (DOCX) [file pone.0182363.s003.docx]

**S3 Table: Description of the stunted-overweighted, stunted non-overweight and non-stunted overweight populations**

|  | **Stunted, non overweight**  **N=120** | **Stunted, overweight**  **N=28** | **Non stunted, overweight**  **N=14** |
| --- | --- | --- | --- |
| Mean HAZ^1^ | -3.12 ± 1.0 | -3.72 ± 0.99 | -0.79 ± 0.96 |
| Mean WHZ^1^ | 0.12 ±1.03 | 2.42 ± 0.78 | 2.51 ± 0.51 |
| Mean zBMI^1^ | 0.44 ± 0.99 | 2.94 ± 0.74 | 2.61 ± 0.50 |
| Females | 42/120 (35%) | 10/28 (36%) | 8/14 (57%) |
| Age (months)^2^  Infant  Toddler  Child | 15 (10; 23)  38/120 (32%)  56/120 (46%)  26/120 (22%) | 16.5 (11.5; 21)  7/28 (25%)  16/28 (57%)  5/28 (18%) | 12 (8; 14)  6/14 (43%)  7/14 (50%)  1/14 (7%) |
| Water source of child  At least sometimes water from well  Running water or from fountain only  Only pure water  Other (breastfeeding etc.) | 29/120 (24%)  69/120 (58%)  15/120 (12%)  7/120 (6%) | 6/28 (21%)  17/28 (61%)  4/28 (14%)  1/28 (4%) | 3/14 (22%)  8/14 (14%)  2/14 (57%)  1/14 (7%) |
| Eating with  Fingers only  Cutlery only  Both | 41/120 (35%)  41/120 (35%)  35/120 (30%) | 12/28 (43%)  10/28 (36%)  6/28 (21%) | 4/14 (29%)  7/14 (50%)  3/14 (21%) |
| Mother lives with family | 112/120 (93%) | 27/28 (96%) | 14/14 (100%) |
| Mother completed at least primary school | 66/120 (55%) | 17/28 (61%) | 9/14 (64%) |

| Socioeconomic score^3^  Lowest income  Middle income  Highest income | 12/120 (10%)  97/120 (81%)  11/120 (9%) | 3/28 (11%)  22/28 (78%)  3/28 (11%) | 1/14 (7%)  9/14 (64%)  4/14 (29%) |
| --- | --- | --- | --- |
| Pathogen found  (Parasite, Bacterium or Virus) | 57/120 (49%) | 14/28 (50%) | 8/14 (57%) |
| Parasite found | 22/120 (18%) | 6/28 (21%) | 1/14 (7%) |
| Virus found | 18/120 (16%) | 2/28 (7%) | 1/14 (7%) |
| Bacterium in culture found | 16/120 (13%) | 2/28 (7%) | 1/14 (7%) |

*Overweight defined as zBMI ≥2

^1^Mean ± standard deviation

^2^Median (Q25, Q75)

^3^ as described in Breurec et al., PNTD 2016
